# Genome-Wide Identification and Characterization of Calcium Metabolism Related Gene Families in *Arabidopsis thaliana* and Their Regulation by *Bacillus amyloliquefaciens* Under High Calcium Stress

**DOI:** 10.3389/fpls.2021.707496

**Published:** 2021-08-11

**Authors:** Jiyi Gong, Tianlong Shi, Yuke Li, Hancheng Wang, Fei Li

**Affiliations:** ^1^The Key Laboratory of Biodiversity Conservation in Karst Mountain Area of Southwest of China, Forestry Ministry, School of Life Sciences, Guizhou Normal University, Guiyang, China; ^2^Key Laboratory of Plant Physiology and Developmental Regulation, School of Life Sciences, Guizhou Normal University, Guiyang, China; ^3^Upland Flue-cured Tobacco Quality and Ecology Key Laboratory of China Tobacco, Guizhou Academy of Tobacco Science, Guiyang, China

**Keywords:** calcium-related gene family, HMM-profile gene search, sequence motif conservation, synteny, transcriptome, *Bacillus amyloliquefaciens*, *Arabidopsis thaliana*

## Abstract

Several gene families involved in calcium signaling have been detected in plants, including calmodulin (CaM), calcium dependent protein kinases (CDPK), calcineurin B-like (CBL) and cyclic nucleotide-gated channels (CNGCs). In our previous study, we demonstrated that *Bacillus amyloliquefaciens* LZ04 (*B. amyloliquefaciens* LZ04) regulate genes involved in calcium stress in *Arabidopsis thaliana* (*A. thaliana*). Here, we aimed to explore the potential involvement of calcium-related gene families in the response of *A. thaliana* to calcium stress and the potential regulatory effects of *B. amyloliquefaciens* LZ04 on these genes. The structure, duplication, synteny, and expression profiles of 102 genes in calcium-related gene families in *A. thaliana* were investigated. Hidden Markov Models (HMMs) and BLASTP were used to predict candidate genes and conserved domains of the candidate genes were confirmed in SMART and NCBI CDD databases. Gene duplications and synteny were uncovered by BLASTP and phylogenetic analysis. The transcriptome expression profiles of candidate genes were investigated by strand-specific sequencing. Cluster analysis was used to find the expression profiles of calcium-related genes families under different treatment conditions. A total of 102 genes in calcium-related gene families were detected in *A. thaliana* genome, including 34 CDPK genes, 20 CNGC genes, 18 CIPK genes, 22 IQD genes, and 10 CBP genes. Additionally, of the 102 genes, 33 duplications (32.35%) and 26 gene pairs including 48 genes (47.06%) were detected. Treatment with *B. amyloliquefaciens* LZ04 enhanced the resistance of *A. thaliana* under high calcium stress by regulating some of the genes in the calcium-related gene families. Functional enrichment analysis revealed that the genes clustered in the 42nd expression profile which may be *B. amyloliquefaciens*-responsive genes under calcium stress were enriched in protein phosphorylation and protein modification process. Transcriptome data was validated by RT-PCR and the results generally corroborated the transcriptome sequencing results. These results may be useful for agricultural improvement in high calcium stress regions.

## Introduction

The adverse effects of abiotic factors on plants in a specific environment are called abiotic stresses, among which drought, flood, and salinity are the most common. Specifically, calcium stress is one of the most crucial abiotic stresses affecting plant growth. Calcium (Ca^2+^) is a critical element in plants and plays a critical role in the response to external stimuli, resulting in a series of protective physiological responses, thereby attenuating the damages caused by the environment. However, excessive calcium concentration in the soil can cause osmotic pressure and interfere with nutrient ion balance leading to the inhibition of plant growth and development. The identification of calcium-related genes in plants can help reveal the physiological mechanisms of plant resistance to abiotic stress. *Arabidopsis thaliana* is a model organism in plants and its genome sequence has been fully studied. Therefore, it is of significance to investigate calcium-related genes and their expression profiles in *A. thaliana* under calcium stress, which can contribute to the improvement of crops.

Several gene families involved in calcium signaling have been detected in plants. Previous studies reported several classes of calcium binding proteins, including Ca^2+^-dependent protein kinases (CDPK), calmodulin (CaM), and CaM-like proteins (CMLs) (Ranty et al., 2006; Yip Delormel and Boudsocq, 2019). CDPKs are encoded by a large gene family and play important role in the resistance against biotic and abiotic stresses, and in plant growth and development (Asano et al., 2012). A previous study reported the roles of *A. thaliana* CDPKs in abiotic stress responses and plant growth regulation (Shi et al., 2018). CDPKs were also reported as key factors driving the pathways that convert calcium signals into physiological responses through a series of phosphorylations (Yip Delormel and Boudsocq, 2019). CaM is responsible for the conversion of Ca^2+^ signals into appropriate responses (Aldon et al., 2018). In plants, CaM mediates the regulation of enzymes, ion channels and other proteins by Ca^2+^. It was also reported that the biosynthesis and signaling of jasmonates (JAs), which are related to changes in intracellular calcium levels, is regulated by CaMs, CMLs, CDPKs and calcineurin B-like proteins (CBLs) (Wang X. et al., 2019). IQD proteins or IQ67-domain proteins belong to a cluster of CaM/CML-target molecules specific to plant and characterized by a common unique domain containing multiple tandem CaM motifs. The IQ67-domain is responsible for plant growth and development through cellular auxin and calcium signaling. Calmodulin-binding proteins (CBPs) are proteins binding with CaM which is implicated in the stress response in plants (Perruc et al., 2004; Virdi et al., 2015). CBL-interacting protein kinase (CIPK) is a family of protein kinases that bind with CBL proteins, which serve as a key factor in signal transduction and stress resistance. Moreover, CBL-CIPK may promote the occurrence of the C/N nutrient response and acquisition of magnesium and iron (Saito and Uozumi, 2020). The cyclic nucleotide-gated channels (CNGCs) are the members of the non-selective cation channel gene families and play an important role in plant development, signal pathway and abiotic stresses. Additionally, CNGCs mediate Ca^2+^ influx (Chiasson et al., 2017) and immune responses (Moeder et al., 2019) in plants.

*Bacillus amyloliquefaciens* belongs to the genus *Bacillus* and has been isolated from different habitats, including animals, plants, food, soil and various environment (WoldemariamYohannes et al., 2020). *B. amyloliquefaciens* is widely found in the soil and can decompose compounds such as invalid calcium, phosphorus, iron and phosphorus that cannot be easily absorbed by plants into their available and usable forms in the soil. Chen and colleagues reported the role of *B. amyloliquefaciens* FZB42 in plant growth and inhibition of plant pathogens (Chen et al., 2007). The volatile organic compounds (VOCs) released by *B. amyloliquefaciens* SAY09 have been reported to alleviate Cd toxicity in *A. thaliana* via increment of auxin biosynthesis (Zhou et al., 2017). Additionally, our previous study reported that *Bacillus amyloliquefaciens* LZ04 promotes the growth of *A. thaliana* in 40 mM CaCl_2_ compared with the corresponding control (Li et al., 2020a). However, the change of the expression level of the calcium-related genes in *A. thaliana* co-cultivated with *B. amyloliquefaciens* has not been investigated.

The members of CDPK, CIPK, CNGC, and CBP gene families have been detected in various plants, including *Triticum aestivum* (Li et al., 2008; Guo et al., 2018; Luang et al., 2018; Wang Y. et al., 2019), *Zea mays* (Hu et al., 2007; Mittal et al., 2017; Hao and Qiao, 2018; Chen et al., 2021), *Oryza sativa* (Zhang T. et al., 2005; Kanwar et al., 2014; Ni et al., 2019; Cui et al., 2020) and *A. thaliana* (Perruc et al., 2004; Jin et al., 2015; Zhou et al., 2016; Wang P. H. et al., 2019). The IQD family has also been reported to respond to abiotic stress in *A. thaliana* (Abel et al., 2005; Wu et al., 2016). However, to the best of our knowledge, the roles of genes in calcium-related gene families from the five gene families mentioned above have not been elucidated completely in *A. thaliana*. The *A. thaliana* genome is crucial for the genome-wide identification of gene families. Unfortunately, how calcium stress is regulated by genes in calcium-related gene families in *A. thaliana* remain unexplored. Therefore, the combination of bioinformatics and transcriptome analysis would systematically reveal the evolutionary relationships, expression profiles and potential functions of members from CDPK, CIPK, CNGC, CBP, and IQD gene families, and may deepen our understanding of the tolerance of *A. thaliana* under high calcium stress.

In this study, a genome-wide analysis of the *A. thaliana* genome identified 102 genes in calcium-related gene families, which were divided into five gene families (CDPK, CIPK, CNGC, CaM, and IQD). The characteristics of these genes, including chromosomal locations, structures, conserved domains, duplications, *cis*-elements, phylogeny and synteny were further investigated. Moreover, the effects of *B. Amyloliquefaciens* LZ04 on the expression of these genes in *A. thaliana* under high calcium stress were also explored.

## Materials and Methods

### Sequence Retrieval and Identification of Calcium-Related Gene Families

The whole genome sequence, CDS sequences, protein sequences and GFF annotation files of *A. thaliana* were downloaded from Ensembl Plants.^1^ The FASTA index file of the whole genome sequence was created by the fadix command in SAMtools. Several studies reported that the members of CDPK, CIPK, IQD, CBP, and CNGC families are associated with the regulation of calcium in *A. thaliana* (Abel et al., 2013; Cheval et al., 2017; Chiasson et al., 2017; Wang P. H. et al., 2019; Lu et al., 2020). In addition, in the present study, a search in the annotation files of our transcriptome data with the keyword “calcium” returned results of genes predominantly belonging to CDPK, CIPK, IQD, CBP, and CNGC gene families. Therefore, we focused on these five genes families. The HMMs of the five gene families were downloaded from Pfam^2^ with the keywords “CDPK,” “CIPK,” “IQ,” “calmodulin,” and “cyclic nucleotide-gated channel” and were used to rescan calcium-related gene families in *A. thaliana*.

Two methods were used to search the calcium-related gene families in *A. thaliana*. Firstly, HMMs were used to predict the potential calcium-related genes and the candidate genes were screened by *E*-values. Secondly, the protein sequences of candidate genes were uploaded on NCBI BLASTP^3^ to obtain the genes with specific conserved domain. The genes with the specific conserved domain of the five gene families were filtered and kept based on the identity values. The conserved domains of calcium-related gene families were further confirmed by MEME suite (Bailey et al., 2009), NCBI’s Conserved Domain Database (CDD) search,^4^ and SMART.^5^ The genes with specific conserved domains were used for further study.

The Expasy ProtParam^6^ was used to investigate the physical and chemical parameters of protein sequences of calcium-associated gene families. The gene structure of candidate genes was visualized by MapChart (version 2.3) (Voorrips, 2002). TBtools (Chen et al., 2020) was used to visualize the motifs and CDS regions of candidate genes.

### *Cis*-Element Analysis

The *cis*-elements in the promoter are considered to be related to the regulation of genes. In order to further explore the potential regulatory network of 102 calcium-related genes, we selected the sequence of 2000bp upstream of the start codon of these genes and used PlantCARE^7^ to obtain *cis*-elements.

### Phylogenetic Analysis and Collinearity Analysis

Phylogenetic analysis of candidate genes was performed by using protein sequences. MEGA (version 10.2.4) (Kumar et al., 2018) was used to perform the phylogenetic analysis. The multiple sequence alignments of candidate genes were completed by MUSCLE. Maximum Likelihood Trees of the aligned sequences were performed by MEGA. The maximum likelihood trees were supported by 1000 bootstrap replicates. The Evolview^8^ was used to visualize the trees.

To uncover the intraspecies microsynteny groups in *A. thaliana*, the MCScanX plugin in TBtools was used to perform collinearity analysis of *A. thaliana*. The circos plots of microsynteny groups were generated by Circos plugin in TBtools. BLASTP was used to identify the repeat sequences based on the CDS sequences of genes in calcium-related gene families in *A. thaliana*. The duplications were obtained based on the following criteria as mentioned in a previous study (Gu et al., 2002): (a) length of the alignable sequence covers 75% of the longer gene; (b) similarity of aligned regions >75%.

### Culture and Stress-Induction Conditions

To confirm the expression of the genes in the calcium-related gene families in *A. thaliana* with high calcium stress, we designed a comprehensive experiment. LB solid medium was used to culture the *B. amyloliquefaciens* LZ04 and temperature was set at 28°C. A single colony was picked and placed in LB liquid medium to prepare the bacterial solution. The *A. thaliana* ecotype Columbia seeds were sown on 0.6% MS medium after sterilization. The separator plates were used in the experiments.

The summary of culture conditions was as depicted in Table 1. Two types of culture media were prepared on plates with a separator: the calcium-free medium and the medium containing 40 mM of CaCl_2_. For the calcium-free medium, 5 ml of 1% MS medium was added to the right and left sides of the separator. For the medium with calcium, in addition to the MS medium added on both sides of the separator as in the calcium-free medium, 20 μL of a stock solution of 2 mM CaCl_2_ was added on both sides of the separator before solidification of the medium to obtain a final concentration of 40 mM of CaCl_2_ in both sides of the corresponding plates. After the preparation of the two types of media, seeding of four uniform-sized seedlings of *A. thaliana* was done on the right side of the plate while seeding of bacterial strains *B. amyloliquefaciens* LZ04 or *E. coli* was done on the 1/4 plate of the left side medium. Then, the plates were cultured at 23°C for 48 h. The *E. coli* strain was used as a control strain to verify the specific effect of *B. amyloliquefaciens* LZ04. Finally, 4 treatment groups were obtained as follows: the control group [0 mM CaCl_2_ on both sides + *E. coli* (left side of the separator)+ *A. thaliana* (right side of the separator)], the LZ04 group [0 mM CaCl_2_ on both sides + LZ04 (left side of the separator) + *A. thaliana* (right side of the separator)], the CaCl_2_ group [40 mM CaCl_2_ on both sides + *E. coli* (left side of the separator)+ *A. thaliana* (right side of the separator)] and the CaCl_2_ + LZ04 group [40 mM CaCl_2_ on both sides + LZ04 (left side of the separator) + *A. thaliana* (right side of the separator)]. Then we collected the root samples of *A. thaliana* grown in the four treatment conditions for whole transcriptome analysis.

**TABLE 1:** Summary of culture conditions indicating the type of media and strains culture in each side of plate separators.

**Type of medium**	**Medium and strains**	**Left side of the plate separator**	**Right side of the plate separator**
Calcium Free Medium	Medium	1% MS medium	1% MS medium
	Bacterial strain	*E. coli*	*B. amyloliquefaciens* LZ04
CaCl2 medium	Medium	1% MS medium + CaCl2 (final concentration of 40 mM CaCl2)	1% MS medium + CaCl2 (final concentration of 40 mM CaCl2)
	Bacterial strain	*E. coli*	*B. amyloliquefaciens* LZ04

### Physiological and Biochemical Indicators of *A. thaliana* Under High Calcium Stress and Strain LZO4 Treatment

The vernier scale was used to evaluate the length of *A. thaliana* roots while the weight of the seedlings was assessed by the dry weight approach. The detection of Na^+^ and K^+^ in was performed with ion chromatography (de Caland et al., 2012). A modified protocol of nitrogen blue tetrazolium (NBT) approach was used for measuring the activity of superoxide dismutase (SOD) whereas the levels of malondialdehyde (MDA) was evaluated by means of the thiobarbituric acid chromogenic approach (Zhang H. et al., 2005). Peroxidase (POD) activity was determined with the guaiacol method (Zhang H. et al., 2005) while H_2_O_2_ content detection followed a previously described method (Wang et al., 2001). Ascorbate peroxidase (APX) activity also followed a previously published protocol (Dalton et al., 1987) whereas catalase (CAT) activity was evaluated by spectrophotical analysis of H_2_O_2_ absorbance at 240 nm (Zhang H. et al., 2005). The detailed information of high calcium stress and strain LZO4 treatment was as described in our previous work (Li et al., 2020a). We thank Professor Jinlin Zhang of Lanzhou University for providing the strain LZ04.

### Transcriptome Analysis

The total RNA of *A. thaliana* root samples was extracted with the Plant RNA Purification Reagent (Invitrogen, Carlsbad, CA, United States). Illumina HiSeq 4,000 was used to perform strand-specific sequencing. The detailed information of the transcriptome analysis was as described in our previous work (Li et al., 2020a).

### Differential Expression Analysis of the *A. thaliana* Root Transcriptome Expression Data

Analysis of variance (ANOVA) was used to perform the differential expression analysis for comparison among the four treatment groups, including LZ04, CaCl_2_, Control, and LZ04 +CaCl_2_ groups. False discovery rate (FDR) was used to perform multiple corrections and was implemented by the R p.adjust() function. Differentially expressed genes (DEGs) were identified if *P* < 0.05. The FPKM gene expression data of genes in the calcium-related gene families were visualized by R “pheatmap” package.^9^

### Cluster Analysis and Functional Enrichment Analysis of Genes in Calcium-Related Gene Families

Short Time-series Expression Miner (STEM) software (Ernst and Bar-Joseph, 2006) was used to perform the cluster analysis of genes in calcium-related gene families. To investigate the biological function of the genes in calcium-related gene families, we performed GO and KEGG analysis using the R “clusterProfiler” package (Yu et al., 2012). The top ten terms of biological process (BP), cellular component (CC), molecular function (MF) and KEGG pathways ranked by *adj.p.value* were visualized.

### Protein-Protein Interaction Network

The string database^10^ was used to construct the protein-protein interaction (PPI) network. The confidence was set at high confidence (0.7).

### Quantitative Real-Time PCR (RT-qPCR)

The transcriptome data was validated by RT-qPCR. Total RNA extraction and purification, reverse-transcription and amplification protocols and conditions conformed with those in our previous work (Li et al., 2020a). The forward and reverse sequences of primers used were as shown in Table 2. GAPDH was used as an internal housekeeping gene; the 2^–ΔΔCt^ approach was used for the relative gene expression calculation and the one-way ANOVA analysis was performed to detect the significance of the differences between groups at a *p*-value cutoff of 0.05 by means of GraphPad Prism 8 software (GraphPad Software, San Diego, California, United States). All qRT-PCR experiments were performed in triplicate.

**TABLE 2:** Primers used in this study.

**Gene ID**	**Gene symbol**	**Forward primer (5′- > 3′)**	**Reverse primer (5′- > 3′)**
AT4G30960	SIP3	GGATACGACGGAGCTAAGGC	GCCACGACTCAAACACACAC
AT2G28260	CNGC15	CACCGGTGTTGTAACCGAGA	AAAGAGAGGCTTACTCGGCG
AT2G30360	SIP4	TGACGACGTCTGATCGCAAA	AGATGGAGGGGCAAAATGGG
AT5G10930	CIPK5	CGAAGACGCTGCTCGTAGAT	AAAGCTAACGGCGGAGTGAA
AT4G36070	CPK18	CCACATTCTCATGCCCACCT	CATGGCGTTTTTGGTGTGGT
AT4G04700	CPK27	ACAACAAAAGACAATGTAAAACCCG	CGGAGAAAGAAGCTGCTGGA
AT3G59690	IQD13	TCCTGCAGTCTGACGATTGG	AACACAAACAACACTCAAAATCATC
AT5G37500	GORK	CGGAATCGTGGGAAGGAGAC	TGGTGGGACACTTCCTTGTG
AT5G62070	IQD23	TTGACGTCGGCGTAAAAGGA	TGGTTTGCTTTGGCTACTTTGG
AT1G61950	CPK19	TCCGGACTGGAAAAACTCGG	TCGGTTTCCGACACATACCC
AT2G17890	CPK16	TCAAGAAACGCAAGAATCCGAA	TCCCCTACGGTAAGCGTGTA
AT4G18700	CIPK12	GTTGGAGGGTTGCCTAGACC	ACCCCTCATGATCTCTGGCT
AT1G01340	CNGC10	ACAATTGTGTTGTGTGTATGTGT	ACGGGACCAGTTGCTTGAAT
AT2G34180	CIPK13	ACAACCGAAACAACAACCCC	AATAAGCTGGTGTCCCGCAA
AT4G04695	CPK31	TCCCTGCACTCCAAATGTCC	AGATTGGTCTTGCACAAGTCTCT
AT2G24270	GAPDH	AGTTGCACTACCAACTGCCTTGCT	AGGTCAACCACGGACACATCAACA

## Results

### Identification of Genes in Calcium-Related Gene Families in *A. thaliana*

To identify potential genes in calcium-related gene families in *A. thaliana*, six HMM profiles were downloaded from the Pfam database, including PF00036, PF00069, PF00612, PF07887, PF07839, and PF00027. SMART and NCBI CDD databases were used to confirm the conserved domains of genes. The conserved domains associated with the sequences were Calmodulin_binding, MSCRAMM_ClfB, PKc_like, CIPK_C, STKc_SnRK3, PLN03192, CAP_ED, Ion_trans, DUF4005, IQ, PRK05901, PTZ00121, PspC_subgroup_2, Adgb_C_mid-like, PTZ00341, COG5022, CBD_MYO6-like, STKc_CAMK, FRQ1, PTZ00184, and EFh_PEF. A total of 98 CDPK candidate genes were identified by PF00036 with an *E*-value lower than 1E-50. The protein sequences of candidate genes were uploaded on NCBI BLASTP and after filtering, 32 CDPK gene sequences with identity ≥ entit were retained. The PF00069 predicted 87 CIPK candidate genes with *E*-value < 1E-70. A total of 23 CIPK genes with identity ≥ 0.70 were filtered and 22 CIPK genes with CIPK conserved domain were confirmed by SMART and NCBI batch CDD. PF00027 predicted 60 CNGC candidate genes (*E*-value < 1E-5) and NCBI BLASTP identified 20 CNGC genes (identity ≥ 0.18) containing the CNGC conserved domain. A total of 44 IQD candidate genes were identified by PF00612 and 22 IQD genes were obtained after the confirmation of the IQD conserved domain. PF07839 and PF07887 were used to predict CBP genes and 15 CBP candidate genes were found. A total of 11 CBP genes were identified by NCBI BLASTP (identity ≥ 0.35) and one CBP gene was removed after confirmation of CBP conserved domain. The detailed information and cutoff of the identification of genes were shown in Supplementary Table 1. In total, we obtained 102 genes belonging to calcium-related gene families (CDPK, CIPK, CNGC, CaM, and IQD).

### Chromosomal Location of Genes in Calcium-Related Gene Families in *A. thaliana* Genome

**FIGURE 1:**
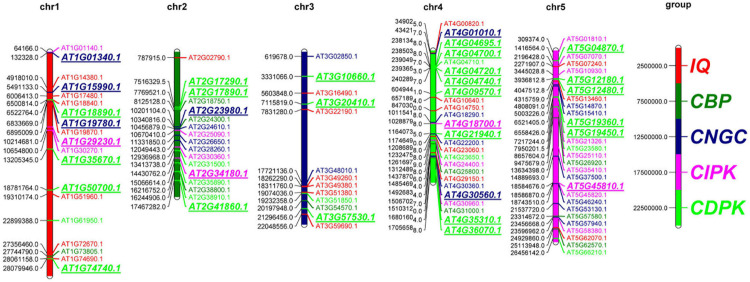
Chromosomal location of the 102 genes in calcium-related gene families in *A. thaliana*. The distribution of the 102 genes on the five chromosomes (chr1-chr5) is presented. Different gene families are marked by different colors. The duplications were marked in italics.

The uneven distribution of genes on the chromosomes may help sequences to be exchanged through recombination or mispairing. We analyzed the distribution of the identified 102 genes in calcium-related gene families on the five chromosomes of *A. thaliana* and found that the distribution of these 102 genes on the chromosomes was not uniform (Figure 1). A total of 53 (52.0%) genes were found to be distributed on chr4 and chr5, while 36 (35.3%) of the remaining genes were distributed on chr1 and chr2; only 13 (12.7%) genes were distributed on chr3. The distribution pattern of genes from different gene families was varied. There were 10 CDPK genes distributed on chr4 and only 4 CDPK genes distributed on chr3. CIPK was mainly distributed on chr5 and the remaining genes were distributed on chr1, chr2, and chr4. CNGC and CBP genes were evenly distributed on the five chromosomes. A total of 7 IQD genes were distributed on chr1, and 6 (27.3%) IQD genes were distributed on chr3. The distribution of genes on chromosomes was also different. The 102 calcium-related genes were mainly distributed at the ends of chr1, chr3, and chr5, but concentrated in the middle and lower parts of chr2 and chr4. The corresponding gene positions of the 102 genes in calcium-related gene families were summarized in Supplementary Table 2. These results indicated that genes in the calcium-related gene families are not uniformly distributed in the genome, which suggests their multifarious roles in *A. thaliana*.

### Characteristics and Structural Analysis of Genes in Calcium-Related Gene Families in *A. thaliana* Genome

We analyzed the coding sequences of the identified 102 genes in calcium-related gene families, including molecular weight (MW), instability index, aliphatic index, grand average of hydropathicity (GRAVY) and so on. As described in Supplementary Table 3, the shortest protein (103 AA) was encoded by the AT3G51380 gene while the longest one (857 AA) was encoded by the AT2G26650 gene. The positive and negative GRAVY values are related to hydrophobicity, with the positive being hydrophobic and the negative being hydrophilic. It should be noted that except for AT1G29230, all proteins encoded by genes in calcium-related gene families were hydrophilic. The aliphatic index of the proteins in CNGC gene family (92.09 ±4.67) was much higher than the average of all families (80.78±11.39). The instability index demonstrated that most of the proteins encoded by these genes were stable. The genome lengths of the 102 genes ranged between 779 and 5,840 bp (Supplementary Table 2).

### Phylogenetic Analysis and Conserved Motifs of the 102 Genes in Calcium-Related Gene Families

**FIGURE 2:**
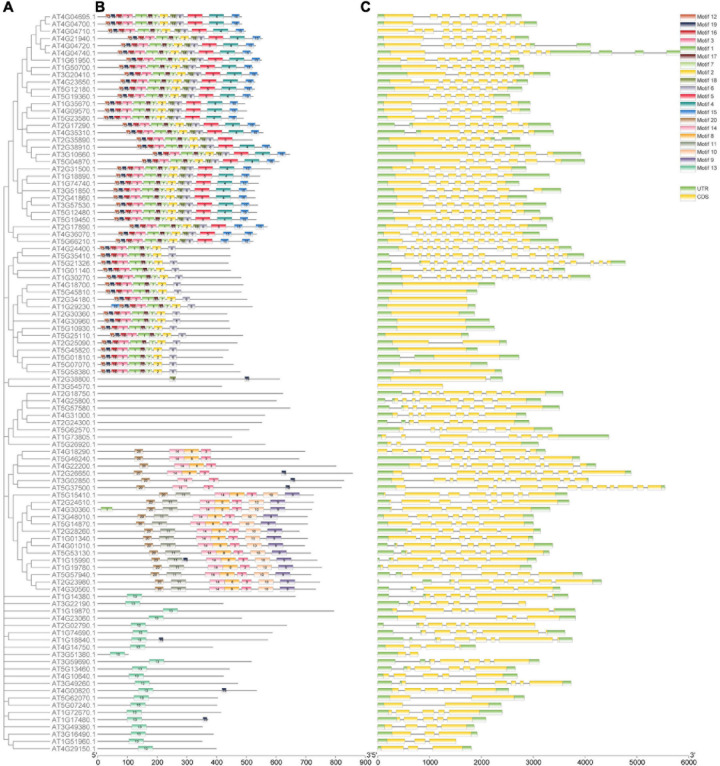
Phylogenetic relationship, structure, and encoded conserved motifs of the 102 genes in calcium-related gene families. **(A)** Phylogenetic tree constructed based on the full-length protein sequences with MEGA-X software. **(B)** Motif composition of proteins associated with calcium. Motifs 1–20 are displayed in various colored boxes. **(C)** Exon-intron structures of the 102 genes in calcium-related gene families. The untranslated regions were colored in green while the exons regions were colored in yellow.

**FIGURE 3:**
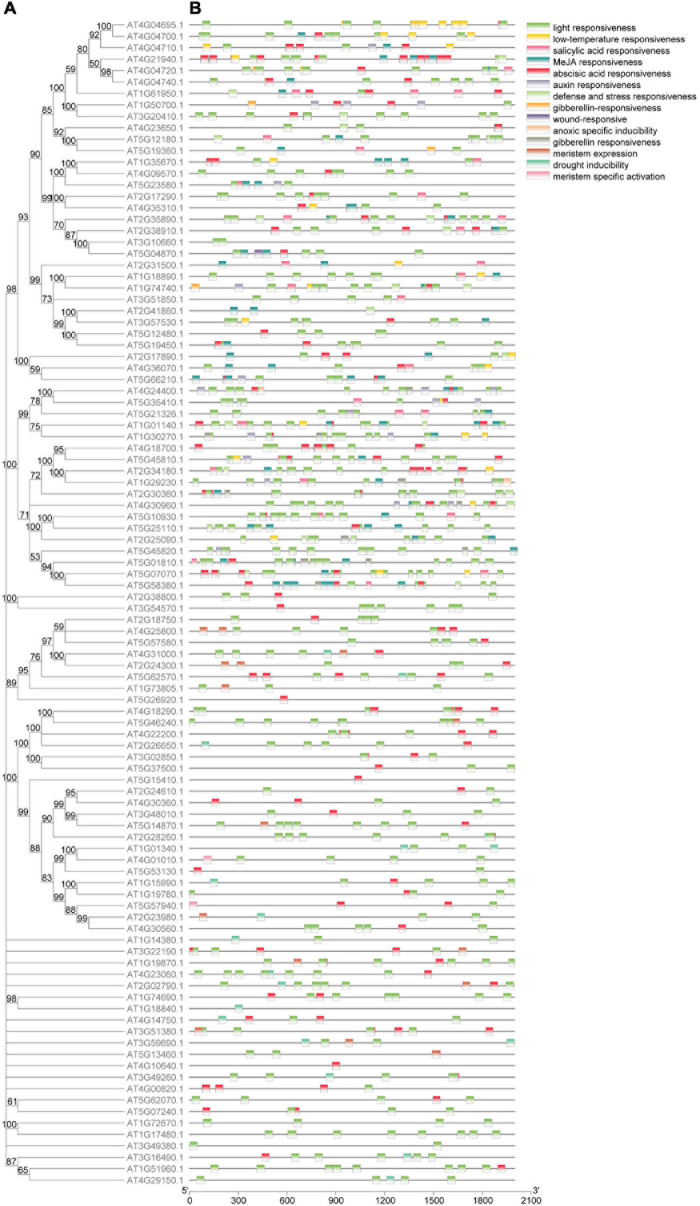
Phylogenetic relationship and *cis*-element of the 102 genes in calcium-related gene families**. (A)** Phylogenetic tree constructed based on the full-length protein sequences with MEGA-X software. Each branch was supported by a bootstrap value. **(B)** The *cis*-element identified by PlantCARE based on the sequence of 2,000 bp upstream of the start codon of the 102 genes in calcium-related gene families.

**FIGURE 4:**
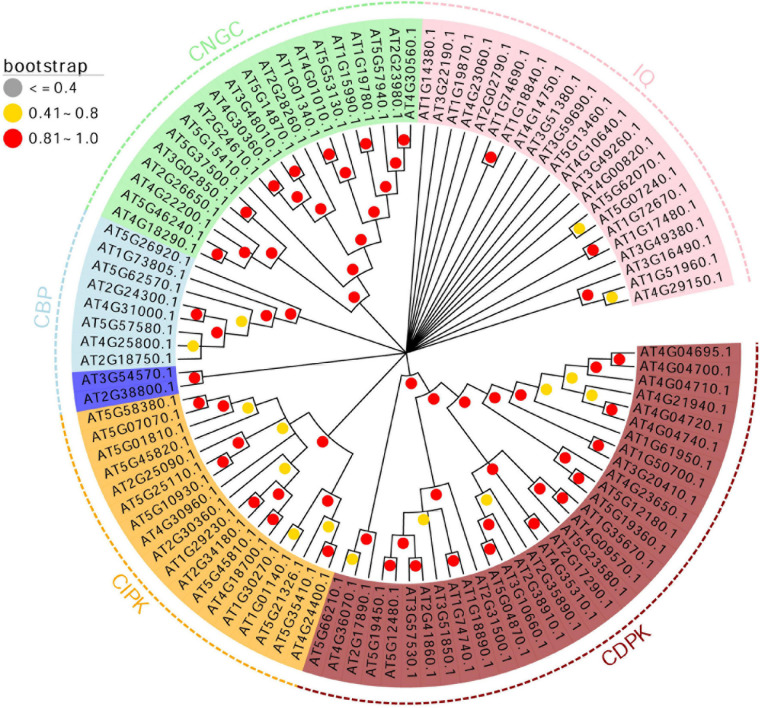
Phylogenetic analysis of the 102 genes in calcium-related gene families. A maximum likelihood phylogenetic tree was constructed based on the 102 genes in calcium-related gene families. The boostrap values (1,000 replicates) are visualized in red (0.81 < bootstrap < 1.0), yellow (0.41 < bootstrap < 0.8) and gray (bootstrap ≤ 0.4).

The 102 genes were divided into five groups based on the phylogenetic analysis (Figures 2A, 3A, 4). To further clarify the potential functions of the 102 genes in *A. thaliana*, we used MEME to identify 20 coding conserved motifs. No motif was detected for genes belonging to the CBP gene family (Figure 2B). The motif analysis showed that the number of motifs in the CDPK gene family was the highest, followed by the CNGC gene family. Genes containing motif1 and motif2 were the most abundant (Supplementary Table 4). The concise hits of conserved domains were PKc_like for motifs 1, 2, 3, 7, and 18, PTZ00184 for motif4, Efh for motif 5 and 15 and CAP_ED, COG5022, PLN03192, and PLN03192 for motifs 10, 13, 14 and 20, respectively (Supplementary Table 4). In order to explore the coding sequences of the 102 calcium-related genes in *A. thaliana*, we compared the number of exons in each gene. As shown in Figure 2C, the average number of exons in the CDPK gene family in *A. thaliana* was greater than the other gene families, followed by the CNGC gene family. Additionally, the encoding sequences of CIPK gene family contained a smaller number of CDS, but a larger CDS region. These results indicated that the number of exons in the 102 genes in calcium-related gene families has increased or decreased during evolution.

### *Cis*-Element Analysis

A total of 15 *cis*-elements were detected, including 653 light responsiveness *cis*-elements, 236 abscisic acid responsiveness *cis*-elements, 148 MeJA responsiveness *cis*-elements, 20 drought inducibility *cis*-elements, and 24 anoxic specific inducibility *cis*-elements (Figure 3B). The most and least *cis*-acting elements found in the promoter regions of the 102 calcium-related genes were light responsiveness *cis*-elements (653) and wound-response *cis*-elements (1), respectively (Supplementary Table 5).

### Gene Duplication and Synteny Analysis of the 102 Genes in Calcium-Related Gene Families

Duplications are important evolutionary events that increase the complexity of the genome. Our data revealed that 33 of the 102 genes in calcium-related gene families (32.35%) were duplicated and unevenly distributed in the five chromosomes of *A. thaliana* (Figure 1 and Supplementary Table 6). We found that the duplications were present in the members of the three gene families (e.g., CNGC, CDPK, and CIPK). Chromosome 4 had the highest number of duplications (containing one CIPK gene, two CNGC genes and eight CDPK genes). In contrast, tree duplications were found on chr3 including three CDPK genes.

**FIGURE 5:**
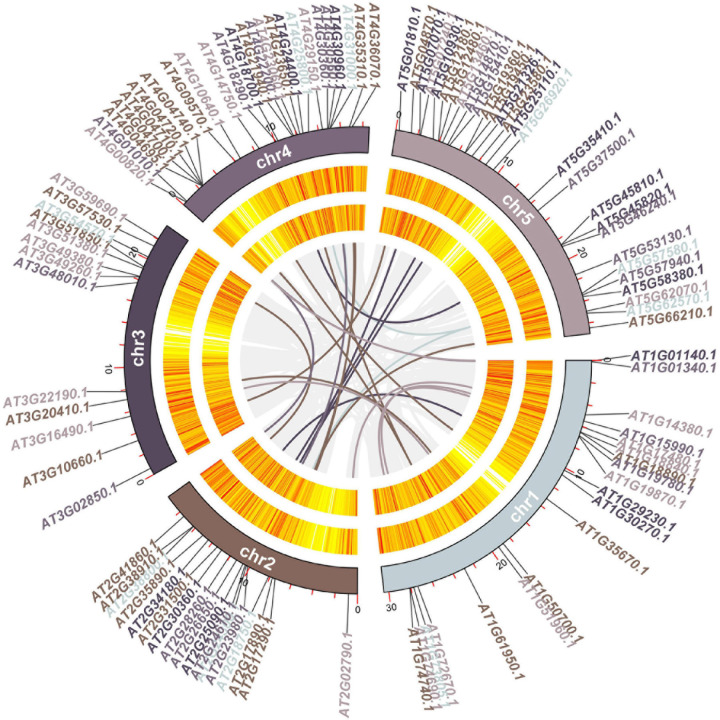
Circos diagram of the 102 genes in calcium-related gene families. Chromosome numbers are provided on each chromosome. The lines in different colors indicate the gene pairs of the 102 genes in calcium-related gene families.

To explore the evolutionary relationships among the 102 genes in calcium-related gene families in *A. thaliana*, we constructed a synteny map for *A. thaliana*. MCScanX methods identified 26 gene pairs including 48 genes among the 102 genes in calcium-related gene families (47.06%) in *A. thaliana* (Figure 5 and Supplementary Table 7). 12 gene pairs were found between the chr1 and other four chromosomes (e.g., chr1, chr2, chr3, and chr4). Specifically, two gene pairs were detected between chr4 and chr5. In addition, 11 gene pairs between chr2 and three other chromosomes (chr3, chr4, and chr5) were detected.

### *B. amyloliquefaciens* Promotes the Adaptability of *A. thaliana* to High Calcium Stress by Regulating Genes in Calcium-Related Gene Families

**FIGURE 6:**
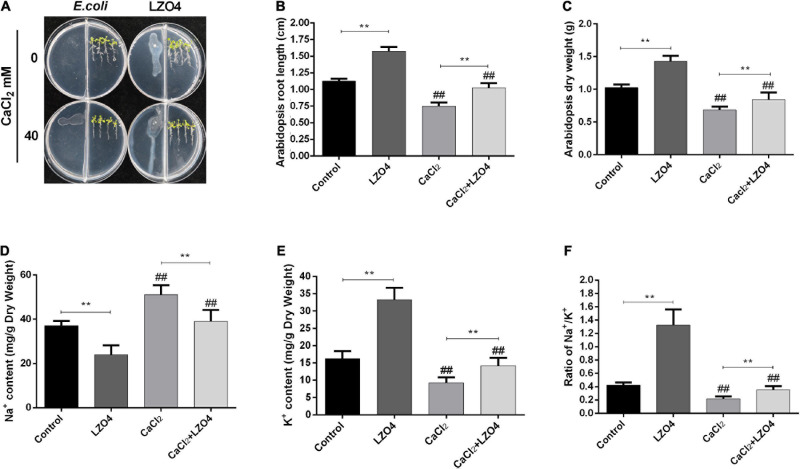
The Adaptation of *A. thaliana* to high calcium stress was increased by *B. amyloliquefaciens* treatment. **(A)** Growth of *A. thaliana* after 48 h. **(B)** The length of *A. thaliana* roots under the four treatments conditions (48 h). **(C)** The dry weight of *A. thaliana* roots. The content of **(D)** Na^+^ and **(E)** K^+^ and **(F)** the ratio of Na^+^/K^+^ in the *A. thaliana* roots. The mean SD (*n* = 5) was used to represent the data. **indicates that significance was found (*P* < 0.01) vs. control or CaCl_2_ groups; ^##^indicates that significance was found (*P* < 0.01) vs. control or LZ04 groups.

**FIGURE 7:**
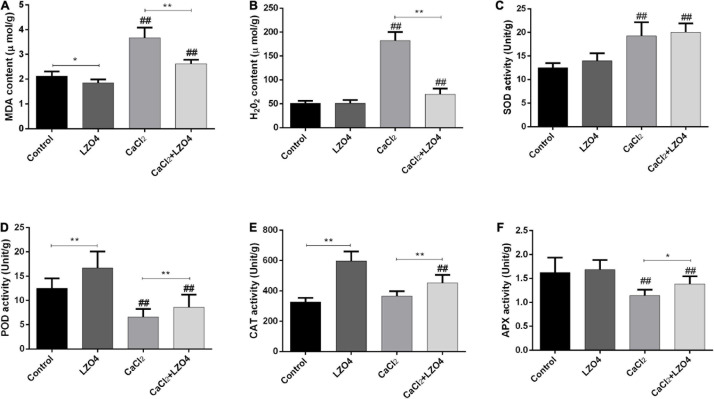
Oxidative stress correlated to products and enzyme activities in *A. thaliana* roots. **(A)** The content of MDA (μmol/g). **(B)** The content of H_2_O_2_ (μmol/g). **(C)** activity of SOD (Unit/g). **(D)** The activity of POD (Unit/g). **(E)** The activity of CAT (Unit/g). **(F)** The activity of APX (Unit/g). The mean SD (*n* = 5) was used to represent the data. * indicates that significant was found (*P* < 0.05) vs. control or CaCl_2_ groups. ** indicates that significance was found (*P* < 0.01) vs. control or CaCl_2_ groups; ^##^indicates that significance was found (*P* < 0.01) vs. control or LZ04 groups.

In order to explore the role of LZ04 in the adaptation of *A. thaliana* to high calcium stress and the possible regulation of genes in calcium-related gene families, we cultured *A. thaliana* in a medium with and without LZ04 in the presence or absence of 40 mM calcium. As shown in Figure 6A, compared with the control group, adding 40 mM CaCl_2_ could significantly inhibit the growth of *A. thaliana* after 48 h. Compared with the control group, inoculation with LZ04 did not affect the growth of *A. thaliana* which grew better in the medium supplemented with CaCl_2_+ LZ04 compared with the CaCl_2_ alone group. The roots of *A. thaliana* were grown extensively in the medium supplemented with LZ04 compared with the control group (Figure 6B). The same phenomenon was also found in the CaCl_2_+LZ04 group compared with the CaCl_2_ group (Figure 6B). Similarly, the dry weight of *A. thaliana* was greater in the LZ04 group compared with the control group (Figure 6C). Compared with the CaCl_2_+LZ04 group, the weight of *A. thaliana* in the CaCl_2_ group was the lowest (Figure 6C). Treatment with *B. amyloliquefaciens* LZ04 decreased the content of Na^+^ under normal and calcium stress conditions (Figure 6D). We detected a higher level of K^+^ in the LZ04 group compared with the control group and a lower level of K^+^ in the CaCl_2_ group compared with the CaCl_2_+LZ04 group (Figure 6E). Similar trends were also found in the ratio of Na^+^/ K^+^ among the four treatment groups and the ratio of Na^+^/K^+^ in the LZ04 and the CaCl_2_+LZ04 groups were higher than the other groups (Figure 6F). The oxidative stress products and enzyme activities (Figure 7) revealed a significant increment of MDA (Figure 7A) and H_2_O_2_ content (Figure 7B) in the group treated with 40 mM CaCl_2_. The presence of 40 mM calcium contributed to the increased level of SOD activity and decreased the activities of POD, CAT, and APX (Figures 7C–F). Except for SOD, the LZ04 with treatment under high calcium stress attenuated the above effects (Figure 7F).

**FIGURE 8:**
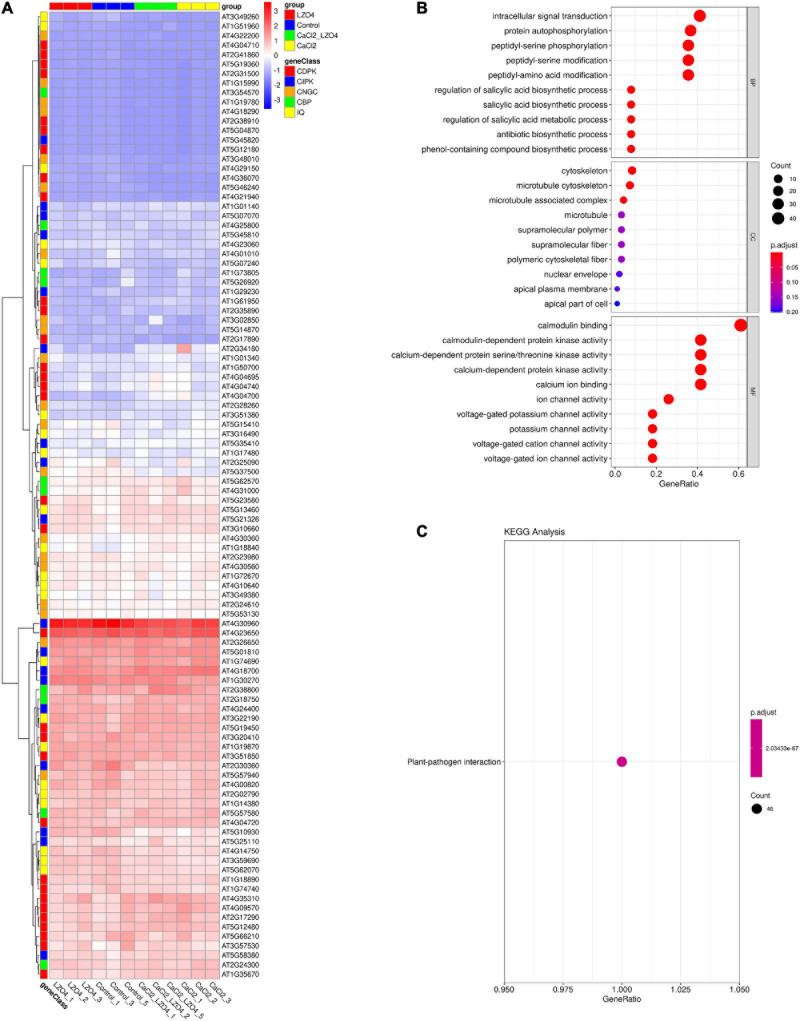
Expression profiles and functional enrichment analysis of genes in calcium-related gene families in the roots of *A. thaliana*. **(A)** The expression profiles of the 102 genes in calcium-related gene families in the whole root of *A. thaliana* were shown in a heatmap. **(B)** The biological function (BP), cellular component (CC), and molecular function (MF) of the 102 genes in calcium-related gene families. **(C)** The KEGG pathways of the 102 genes in calcium-related gene families. The top 10 terms of the GO and KEGG (sorted by *p*.ajd.value) were selected for visualization. The size and color of the dots were related to the number of genes and the *p.adj.value*, respectively.

After confirming the effect of calcium stress and *B. amyloliquefaciens*, to investigate the potential functions of the 102 genes in the pathways associated with calcium in *A. thaliana*, we confirmed the expression of the 102 genes in the *A. thaliana* root with high calcium stress based on transcriptome data. Overall, the expression profile of the 102 genes in calcium-related gene families was shown in Figure 8A. We found that the 102 genes in calcium-related gene families were enriched in the pathways of intracellular signal transduction, cytoskeleton, calmodulin binding, calmodulin-dependent protein kinase activity, voltage-gated potassium channel activity and so on (Figure 8B and Supplementary Table 8). Additionally, the enriched KEGG pathway of the 102 genes was plant-pathogen interaction (Figure 8C). The PPI network indicated that genes in the calcium-related gene families were interconnected and some of them formed a solid block (Supplementary Figure 1). There were also interactions between genes of different gene families (Supplementary Figure 1).

**FIGURE 9:**
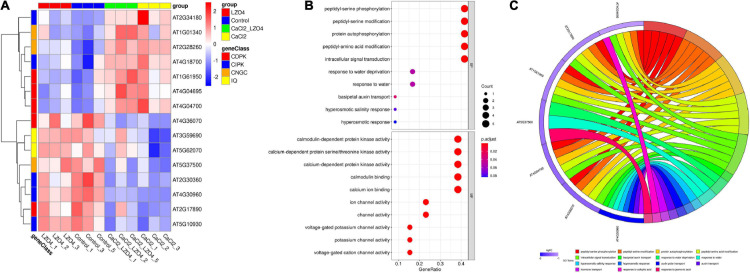
Differential expression analysis and functional enrichment analysis of genes in calcium-related gene families in the whole root of *A. thaliana***. (A)** The expression profiles of DEGs in calcium-related gene families in whole root of *A. thaliana* were shown in a heatmap. **(B)** The biological function (BP) and molecular function (MF) of DEGs associated with calcium. **(C)** The GO enrichment of DEGs in calcium-related gene families. The top 15 terms (sorted by *p.adj.value*) were shown in the circos plot. The top 10 terms of the GO and KEGG results (sorted by *p*.ajd.value) were selected for visualization. The size and color of the dots were proportional to the number of genes and the *p.adj.value*, respectively.

ANOVA was used to reveal the differentially expressed genes (DEGs) in the 102 genes among the four treatment groups and the results was summarized in Supplementary Table 9. We found 15 DEGs among the four treatment groups (Figure 9A and Supplementary Table 10). The heatmap revealed that 7 of the 15 DEGs (46.67%) were under-expressed in LZ04 and Control groups while the remaining 8 genes were highly expressed in these groups (Figure 9A). The enriched pathways of the DEGs were peptidyl-serine phosphorylation, calmodulin-dependent protein kinase activity and calmodulin binding (Figures 9B,C). The top 15 terms (ranked by *p.adj.value*) of the GO analysis showed that AT4G04695, AT2G17890, AT1G61950, AT4G04700, and AT4G36070 were related to the peptidyl-serine phosphorylation and intracellular signal transduction (Figures 9B,C).

**FIGURE 10:**
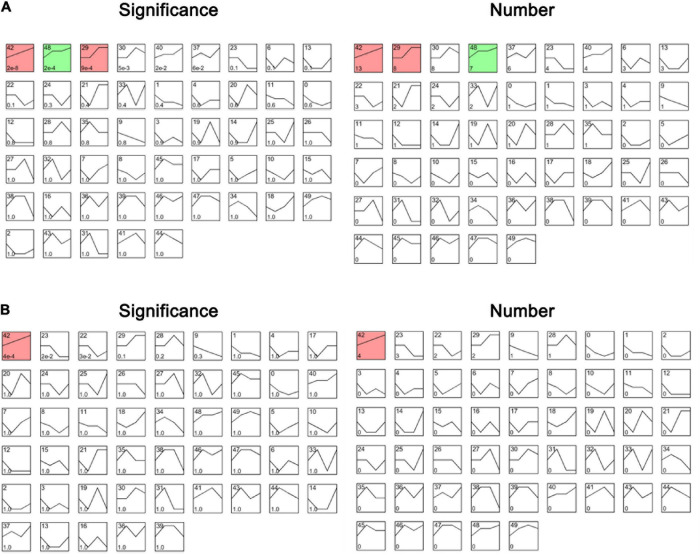
The profiles of cluster analysis based on the expression profiles in the whole root of *A. thaliana*. **(A)** The profiles of cluster analysis by STEM based on the 102 genes in calcium-related gene families ranked by significance and number of genes. **(B)** The profiles of cluster analysis by STEM based on the DEGs associated with calcium ranked by significance and number of genes. The numbers 0–50 in the upper left refers to a serial number of profiles; The number of genes was shown in the lower left; The significance of each profile was described by *P*-value.

To identify the genes related to the four groups (Control, LZ04, CaCl_2_, and CaCl_2_+LZ04) associated with calcium stress, we performed cluster analysis using gene expression profiles. Based on the expression profiles of the 102 genes in calcium-related gene families, three profiles including 28 genes were identified as significant among the four groups (*P* < 0.05) (Figure 10A). Profile 42 contained 13 genes and was associated with the protein phosphorylation, protein modification process and cellular protein metabolic process (Supplementary Table 11). Using expression profiles of 15 DEGs mentioned above, we detected one significant profile among the four groups, which contained four genes (AT1G01340, AT1G61950, AT2G28260, and AT4G04695) (Figure 10B and Supplementary Table 12). The PPI network of the DEGs also showed solid interactions among these genes (Supplementary Figure 2). The most solid interactions were found among the CPK16, CPK18, CPK19, CPK27, and CPK31 genes and the interacteractions among genes from different gene families were also recorded (Supplementary Figure 2).

### RT-qPCR Validation of Gene Expression

**FIGURE 11:**
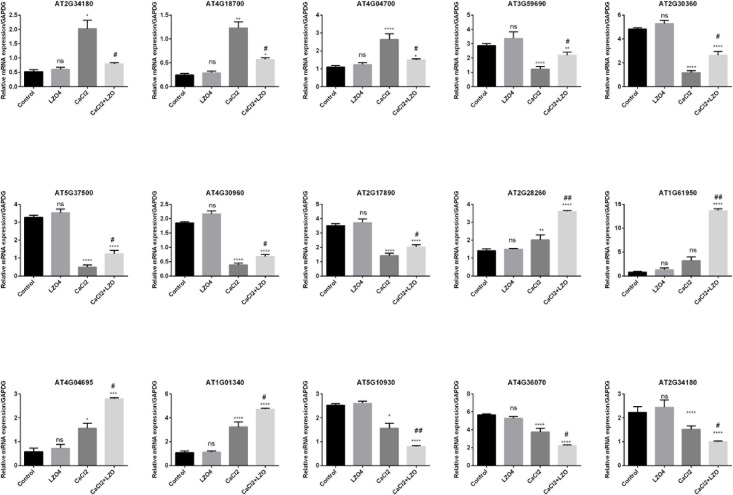
RT-qPCR validation of the expression profiles differentially expressed genes in calcium-related gene families in the whole root of *A. thaliana* under calcium stress and treated with or without *B. amyloliquefaciens*. *p < 0.05, **p < 0.01, ***p < 0.001, ****p < 0.0001 and ns = non-significant compared with control group. ^#^*p* < 0.05, ^##^*p* < 0.01 compared with CaCl_2_ group.

To validate the expression levels of the 15 genes identified as those differentially expressed among the four treatment groups, these genes were selected for RT-qPCR analysis (Figure 11). Compared with the Control group, the expression levels of AT2G34180 (CIPK13), AT4G18700 (CIPK12), and AT4G04700 (CPK27), significantly upregulated in the CaCl_2_ group compared to the control group, was reversed by the LZ04 treatment, but no significant difference was observed between the LZ04 alone and the Control groups (Figure 11). In addition, the expression of genes such as AT3G59690 (IQD13), AT2G30360 (SIP4), AT5G37500 (GORK), AT4G30960 (SIP3), and AT2G17890 (CPK16) were significantly downregulated in the CaCl_2_ group compared to the control group, but this downregulated expression was reversed by the treatment of *A. thaliana* under high calcium stress by LZ04 (Figure 11). Moreover, the expression levels of AT2G28260 (CNGC15), AT1G61950 (CPK19), AT4G04695 (CPK31), AT1G01340 (CNGC10), and AT1G01340 (CNGC10), upregulated in the CaCl_2_ group compared to the control group was further promoted by LZ04 treatment. The expression levels of AT5G10930 (CIPK5), AT4G36070 (CPK18), and AT5G62070 (IQD23) were all downregulated in the CaCl_2_ group, but the LZ04 treatment was unable to reverse this trend (Figure 11). Thus, in general, similar trends in the expression of the 15 differentially expressed genes were found between the RT-PCR and transcriptome sequencing results (Figure 11). In addition, the results of RT-PCR results indicated that the 5 gene families may play significant roles in the response of *A. thaliana* to high calcium stress and in the interaction of this plant species with *B. amyloliquefaciens* which improves the resistance of *A. thaliana* to high calcium stress (Figure 11).

The above results indicated that the calcium-related gene families play significant functions in the adaptation of *A. thaliana* to high calcium stress and in the regulatory effect of LZ04 on this process.

## Discussion

The genes in calcium-related gene families have been detected in various plant species. In this study, we identified the candidate genes in calcium-related gene families in *A. thaliana* and uncovered their distribution, structures, conserved domains, *cis*-elements, duplication and synteny relationships. A total of 102 genes in five calcium-related gene families (CDPK, CIPK, CNGC, CBP, and IQD) were identified through HMM and NCBI BLASTP. Furthermore, we confirmed the conserved domains of these genes via SMART and NCBI CDD. We identified 32 CDPK genes in *A. thaliana*, which was consistent with the results of previous studies (Cheng et al., 2002; Hrabak et al., 2003) in which 34 CDPK genes were detected in *A. thaliana* genome. A total of 20 CNGC genes, 18 CIPK genes, 22 IQD genes and 10 CBP genes were identified in this study. In *A. thaliana* genome, 20 CNGC genes (Mäser et al., 2001) and 26 CIPK genes (Kolukisaoglu et al., 2004) were identified, respectively. CBP genes are not conserved in plants and animals; a total of 27 CBP genes were reported in the *A. thaliana* genome by Reddy and colleagues (Reddy et al., 2002). Similar to our study, Abel and colleagues identified 33 IQD1-like genes in *A. thaliana* (Abel et al., 2005). The above differences may be caused by the different *E*-values used in HMM profilers as well as the manual checking of gene domains.

According to the phylogenetic analysis, the 102 genes in calcium-related gene families were divided into five clusters, including CDPK, CIPK, CNGC, CBP, and IQD. Except for the IQD and CBP gene families, the members of CDPK, CIPK, and CNGC gene families were clustered into corresponding gene family clusters, suggesting these genes were evolutionary conserved. CBP genes were divided into two groups including 2 and 8 CBP genes. The remaining genes belonged to the IQD gene family and the bootstrap value of most branches related to IQD was lower than 0.4. This unique pattern of genes in calcium-related gene families indicates that although these genes are related to calcium, the differences in their coding sequences are significant.

To uncover the characteristics of gene structures and genome evolution of the 102 genes in calcium-related gene families, we performed synteny analysis in *A. thaliana*. Consequently, 26 gene pairs including 48 genes were detected in synteny blocks, suggesting the syntenic regions between the 102 genes in *A. thaliana* genome were conserved. An increasing number of studies have suggested that the development of gene families and corresponding mechanisms involved in genome evolution are associated with gene duplication events (Magadum et al., 2013; Panchy et al., 2016). In this study, we identified 33 duplication events from five gene families in *A. thaliana* genome. These results indicated the presence of variations in gene replications and conserved evolutionary sequences, which is consistent with a previous study (Lockton and Gaut, 2005). The conserved domains of the protein sequences of calcium-related genes were investigated in *A. thaliana*. Compared with the other two gene families, the coding sequences of CDPK, CIPK, and CNGC genes contain more exons, which may be related to the conservative nature of the replication of these genes which regulates the gene structure. This result is consistent with the results in previous studies (Xu et al., 2012; Wang et al., 2013). Although IQD and CBP genes are related to CaM and calcium signaling in *A. thaliana*, these genes were different in the coding sequences and corresponding signaling pathways, and were grouped into different classes according to phylogenetic analysis performed in this study. The motifs detected by MEME revealed the motif compositions in different genes in calcium-related gene families. For instance, coding sequences of CDPK genes and CIPK genes contained motif1, motif2, motif3, motif6, motif7, motif12, and motif 19, but motif4, motif5, motif15, and motif18 were lost in CIPK genes. Similarly, except for motif 3, the motifs detected in the other gene families were unique to each. These differences may contribute to the diverse functions of members of these gene families. In addition, some conserved domains were found to be commonly distributed on the 20 identified motifs in calcium-related family members, and the order of the 20 motifs on proteins were similar. These conserved domains on the protein sequencing and the similar order of motifs indicated that the conserved domains and the disposition of motifs is vital for the activity of the proteins encoded by members of calcium-related gene families. We noticed that genes within the same phylogenetic subgroup shared almost the same genetic structures, lengths, and similar compositions in conserved patterns. In addition, the *cis*-acting elements detected in the promoters of these genes were also phylogenetically similar. These characteristics of the genes in the families of genes-related to calcium in *A. thaliana* therefore indicate their evolutionary relationships.

In this study, we confirmed that LZ04 is involved in the adaptation of *A. thaliana* to high calcium stress. We found that adding LZ04 had no impact on the growth of *A. thaliana* root, but enhanced the resistance of *A. thaliana* under high calcium stress. Na^+^, being a non-essential element, can accumulate in plants. The excessive accumulation of Na^+^ is detrimental in the sense that it can lead to the efflux of K^+^ induced by the efflux of Na^+^ and a subsequent imbalance of cellular homeostasis and oxidative stress, the extreme consequences of which are the cessation of growth and death of plants. Studies have shown that Na^+^ efflux can interfere with Ca2^+^ and K^+^ ions and that calcium supplementation improves the Na^+^/K^+^ balance and is fundamental for the adaptation of plants to salt stress (Rahman et al., 2016). Here, on the contrary, we showed that calcium stress destroyed the Na^+^/K^+^ imbalance. However, we observed that co-cultivating *A. thaliana* with LZ04 led to a lower Na^+^ and a higher K^+^ ion content in *A. thaliana* root. Thus, we speculated that LZ04 may regulate the Na^+^/K^+^ ion balance of *A. thaliana* under calcium stress conditions. The increase of ROS production is a detrimental factor leading to the release of cytotoxic factors such as MDA and H_2_O_2_ and is a key index used to determine the effect of stress on plant and their resistance ability. Under high calcium stress, the level of reactive oxygen species (ROS) and oxidative pressure in *A. thaliana* cells increased, which corroborated the findings of several studies reporting that the level of ROS increases in plants under high calcium stress (Choudhury et al., 2017), resulting in high oxidative stress and activation of genes responsible for ROS (Hasanuzzaman et al., 2020). Consistent with the previous works (Amor et al., 2010; Abd_Allah et al., 2017), we observed an increase of cytotoxicity and oxidative stress markers in the CaCl_2_ and CaCl_2_+LZ04 groups compared with corresponding control, suggesting that LZ04 may help *A. thaliana* tolerate high calcium stress induced by CaCl_2_. These observations signposted that LZ04 promoted the resistance and adaptation of *A. thaliana* to high calcium stress by regulating oxidative stress and lipid peroxidation in the high calcium-stressed plants. This study corroborated with our previous work (Li et al., 2020a) showing that the interaction between plants and microorganism is beneficial for plant to resist against numerous pathogens and biotic as well as abiotic stress conditions. Similarly to our findings, a large number of studies (Qin et al., 2017; Wang et al., 2021) have shown that *B. amyloliquefaciens* strains are rhizosphere-promoting bacteria and that inoculation of these strains can promote plant growth, prevent soil-borne diseases and improve plant resistance to stress. As demonstrated in our previous review (Li et al., 2019b) and research works (Li et al., 2019a, 2020a,b), some of bacillus species are able to produce volatile organic compounds (VOCs) which affect the changes of metabolism in their symbiotic plants. Thus, in the present study, since the LZ04 and *A. thaliana* were cultivated on the medium separated by a separator, we stipulated that the mechanism of the effect of LZ04 on *A. thaliana* may be through the production of VOCs which may probably affect gene regulation and phenotypic changes in *A. thaliana*.

The role of genes in the calcium-related family in the response of plants to calcium stress is not clear. Previous studies have shown that the genes involved in calcium signaling play an important role in biotic and abiotic stresses. Up to date, only our previous study (Li et al., 2020a) gave an insight in the mechanism underlying the response of *A. thaliana* to calcium and the role of its interaction with *B. amyloliquefaciens* in the counteracting effect through lncRNA-miRNA-mRNA networks. Here, we focused on the expression of genes in calcium-related gene families in the roots of *A. thaliana* under high calcium stress and the effect of LZ04 on these genes were also explored. At the transcriptome sequencing level, we found that high calcium stress induced significant changes in the expression of calcium-related gene families in the roots of *A. thaliana*. The significant expression profile in the 102 genes in calcium-related gene families was the 42nd profile and the functional enrichment analysis uncovered that the 13 genes detected in the 42nd profile were enriched in the pathways of protein phosphorylation, protein modification process and cellular protein metabolic process. Phosphorylation is a common process that is activated in plants under salt stress. The regulation of calcium-dependent phosphorylation systems was reported to enable plants to grow and acquire tolerance against abiotic stresses (Saito and Uozumi, 2020). Similar to the findings that autophosphorylation may correlate to Ca^2+^ sensitivity of some members of the CDPK gene family, we detected three CDPK genes (AT1G61950, AT4G04695, and AT4G04700) which were down-expressed in LZ04 and control groups compared with groups under high calcium stress. CIPK proteins are activated by calcineurin B-like proteins (CBLs) and phosphorylated loop region located in the kinase domain (Tang et al., 2020), and are involved in the salinity tolerance mechanisms (D’Angelo et al., 2006). The members of the CNGC gene family were reported to undergo phosphorylation and post-translational modifications (Kakar et al., 2017), and the phosphorylation of CNGC was believed to be responsible for defense signaling (Jarratt-Barnham et al., 2021). Therefore, we speculated that LZ04 attenuates the effects of high calcium stress on *A. thaliana*, and related genes may be involved in this process by the regulation of calcium-dependent phosphorylation systems. Here, we also demonstrated that the calcium-related gene families are deregulated in stress induced by high calcium concentration. Specifically, the results of RT-PCR showed that under calcium-related stress, the level of expression of some genes was upregulated while the expression of other genes was downregulated. For the down-regulated genes, LZ04 bacteria induced or reversed the effect of calcium-related stress on gene expression. These results showed that genes in calcium-related gene families play an important role in the response of *A. thaliana* to stress. In addition, these genes play a major role in the mechanisms induced by the interaction of the bacterial strain LZ04 with *A. thaliana* to induce the latter’s resistance to calcium stress. Another important observation is that in the same family, some genes were inhibited by calcium stress while the expression levels of others were promoted. Similarly, although LZ04 allowed the resistance of *A. thaliana* to calcium stress, in some cases its effect promoted the effect of calcium stress on the expression of these genes, while in other cases the opposite effect was observed. These results suggest that within the same family of genes, genes did not respond in a similar way to stress and to the corrective effect of LZ04. These observations were made for all the family genes studied. These differences may be the consequence of the differences in chromosomal location, gene structure and the composition of conserved domains in the proteins encoded by these genes. In the present study, we observed the presence of a certain number of conserved domains in the sequences of the genes in calcium-related gene families, in particular the domains of Calmodulin_binding, MSCRAMM_ClfB, PKc_like, CIPK_C, STKc_SnRK3, PLN03192, CAP_ED, Ion_trans, DUF4005, IQ, PRK05901, PTZ00121, PspC_subgroup_2, Adgb_C_mid-like, PTZ00341, COG5022, CBD_MYO6-like, STKc_CAMK, FRQ1, PTZ00184, and EFh_PEF. Previous studies showed that these domains play a key role in the calcium-mediated signaling pathway (Schmidt et al., 2011; Halling et al., 2016; Mohanta et al., 2017). These domains serve as a point of attachment between the substrate and the targets, allowing signal transmission (Schmidt et al., 2011; Halling et al., 2016; Mohanta et al., 2017). We state here, given the role of genes in the calcium-related gene families, that the above domains play an important role in the functioning of the proteins encoded by these genes. In view of such observations, we can say that our work revealed the genes in calcium-related gene families in *A. thaliana* and their importance in calcium stress as well as in the resistance facilitated by the interaction with LZ04. The action of LZ04 on the above may be through the productions of VOCs which may stimulates the deregulation of gene expression in *A. thaliana* leaves and subsequently in the roots. The VOCs may also bind to the protein domains and change their activities, leading to the shift of their functions.

In summary, the present work reports that the genes of calcium-related gene families are involved in the response of *A. thaliana* to high calcium stress and their expression are also influenced after the treatment of *B. amyloliquefaciens*. We identified, and extensively analyzed some of calcium-related gene families such as CDPK, CIPK, CNGC, CBP, and IQD and extensively showed the chromosomal localization, duplication, structure, conserved motifs, phylogeny, *cis*-elements of genes in calcium-related gene families. More importantly, we report that the effect of *B. amyloliquefaciens* LZ04 treatments to *A. thaliana* seedlings provide tolerance under high calcium concentration. The use of *B. amyloliquefaciens* LZ04 opens new promising windows for further research and hence has agricultural importance.

## Data Availability Statement

The original contributions presented in the study are included in the article/Supplementary Material, further inquiries can be directed to the corresponding author/s. The transcriptome data was deposited in the China National GeneBank DataBase (CNGBdb) under the project accession number CNP0000745. The direct link for accessing the data is: https://db.cngb.org/search/project/CNP0000745/.

## Author Contributions

JG and TS analyzed the data, wrote, and revised the manuscript. YL and HW contributed to the data analysis. FL, JG, and TS contributed to the conceptualization of the study and funding acquisition. All the authors have read and approved the final version of the submitted manuscript.

## Conflict of Interest

The authors declare that the research was conducted in the absence of any commercial or financial relationships that could be construed as a potential conflict of interest.

## Publisher’s Note

All claims expressed in this article are solely those of the authors and do not necessarily represent those of their affiliated organizations, or those of the publisher, the editors and the reviewers. Any product that may be evaluated in this article, or claim that may be made by its manufacturer, is not guaranteed or endorsed by the publisher.

## Footnotes

1
http://plants.ensembl.org/index.html

2
https://blast.ncbi.nlm.nih.gov/Blast.cgi?PROGRAM=blastp&PAGE_TYPE=BlastSearch&BLAST_SPEC=&LINK_LOC=blasttab

3
http://pfam.xfam.org/

4
https://www.ncbi.nlm.nih.gov/Structure/bwrpsb/bwrpsb.cgi

5
http://smart.embl.de/

6
https://web.expasy.org/protparam/

7
http://bioinformatics.psb.ugent.be/webtools/plantcare/html/

8
https://evolgenius.info/evolview-v2/

9
https://cran.r-project.org/web/packages/pheatmap/index.html

10
https://string-db.org/

## Supplementary Material

Supplementary Figure 1 Protein-protein interaction network constructed based on the 102 genes from the calcium-related gene families.

Supplementary Figure 2 Protein-protein interaction network constructed based on the fifteen differentially expressed genes from the calcium-related gene families.

Supplementary Table 1 The detailed information of the cutoff used in the identification of 102 genes in calcium-related gene families.

Supplementary Table 2 The gene location and lengths of the 102 genes in calcium-related gene families identified in *A. thaliana.*

Supplementary Table 3 The characteristics of the 102 genes in calcium-related gene families in *A. thaliana.*

Supplementary Table 4 The summary of motifs in the 102 genes in calcium-related gene families.

Supplementary Table 5 The *cis*-element of the 102 genes in calcium-related gene families identified in *A. thaliana*.

Supplementary Table 6 The summary of the duplications of the 102 genes in calcium-related gene families *detected* in *A. thaliana*.

Supplementary Table 7 The gene pairs and the corresponding position of the 102 genes in calcium-related gene families in *A. thaliana.*

Supplementary Table 8 The detailed information of pathways enriched in the 102 genes in calcium-related gene families.

Supplementary Table 9 The results of ANOVA based on the expression profiles of all genes in *A. thaliana* root.

Supplementary Table 10 The detailed information of pathways enriched in DEGs associated with calcium.

Supplementary Table 11 The detailed information of cluster analysis based on the expression profiles of 102 genes in calcium-related gene families.

Supplementary Table 12 The detailed information of cluster analysis based on the expression profiles of DEGs among the four treatment groups.
